# Tobacco Smoking Surveillance: Is Quota Sampling an Efficient Tool for Monitoring National Trends? A Comparison with a Random Cross-Sectional Survey

**DOI:** 10.1371/journal.pone.0078372

**Published:** 2013-10-23

**Authors:** Romain Guignard, Jean-Louis Wilquin, Jean-Baptiste Richard, François Beck

**Affiliations:** 1 Scientific Affairs Department, National Institute for Health Promotion and Health Education (INPES), Saint-Denis, France; 2 Cermes3 - Cesames Team (Research Centre Medicine, Sciences, Health, Mental Health, Health Policy), CNRS UMR 8211, Institut National de la Sante et de la recherche Medicale U988, University of Paris Descartes, Sorbonne Paris Cité, EHESS, Paris, France; University of Pennsylvania, United States of America

## Abstract

**Objectives:**

It is crucial for policy makers to monitor the evolution of tobacco smoking prevalence. In France, this monitoring is based on a series of cross-sectional general population surveys, the Health Barometers, conducted every five years and based on random samples. A methodological study has been carried out to assess the reliability of a monitoring system based on regular quota sampling surveys for smoking prevalence.

**Design / Outcome Measures:**

In 2010, current and daily tobacco smoking prevalences obtained in a quota survey on 8,018 people were compared with those of the 2010 Health Barometer carried out on 27,653 people. Prevalences were assessed separately according to the telephone equipment of the interviewee (landline phone owner vs “mobile-only”), and logistic regressions were conducted in the pooled database to assess the impact of the telephone equipment and of the survey mode on the prevalences found. Finally, logistic regressions adjusted for sociodemographic characteristics were conducted in the random sample in order to determine the impact of the needed number of calls to interwiew “hard-to-reach” people on the prevalence found.

**Results:**

Current and daily prevalences were higher in the random sample (respectively 33.9% and 27.5% in 15-75 years-old) than in the quota sample (respectively 30.2% and 25.3%). In both surveys, current and daily prevalences were lower among landline phone owners (respectively 31.8% and 25.5% in the random sample and 28.9% and 24.0% in the quota survey). The required number of calls was slightly related to the smoking status after adjustment for sociodemographic characteristics.

**Conclusion:**

Random sampling appears to be more effective than quota sampling, mainly by making it possible to interview hard-to-reach populations.

## Introduction

Tobacco smoking constantly decreased in France between 1970 and 2005. Nevertheless, the most recent survey carried out in 2010 at a national level revealed an increase compared with 2005, non-significant for men, but significant for women, especially concerning those aged 45–64 [1]. On the whole, tobacco smoking prevalence has increased from 31.5% in 2005 to 33.7% in 2010 in 15–75 years-old, and the prevalence of daily smoking from 27.1% to 29.1% in the same time. Taking into account the huge efforts initiated in France in the late 1970s and reinforced in 1991 (Evin Law) in tobacco control policies, the last being large price increases between 2002 and 2004 and smoke-free laws in public places in 2007-2008, it reveals crucial for policy makers to monitor the evolution of these indicators.

The acknowledgement of this evolution is based on a series of multi-thematic repeated cross-sectional general population surveys, the Health Barometers of the French Institute for Health Promotion and Health Education (*Institut National de Prévention et d’Education pour la Santé - INPES*). These surveys, conducted every five years, are based on random samples of about 30,000 people. A random survey carried out properly is usually considered more accurate than a quota survey since it covers more socially diverse and representative populations. Furthermore, it is known that tobacco smoking depends on social background. However, since the methodology of random surveys is complex, and due to economic and organizational reasons, it is difficult to conduct this kind of survey more frequently, and quotas surveys have sometimes been considered as an acceptable alternative to probability sample surveys [2]. Consequently, a methodological study has been carried out by INPES to assess the reliability of a monitoring system based on less costly regular quota sampling surveys for tobacco smoking prevalence, despite their limits.

## Methods

### Ethics statement

All the data were analyzed anonymously. Participants provided their verbal informed consent to participate in the study at the beginning of the questionnaire. Interviewers keyboarded the participant’s consent as part of the computer-assisted telephone interviews (CATI). For participants under 18, verbal consent was obtained from the head of the family in the same way. The population-based surveys process and the consent procedure were approved by the French individual data protection authority (Commission nationale de l’informatique et des libertés [CNIL]), that considered that written consent was not requested for such surveys.

### Health Barometer

The 2010 Health Barometer fieldwork was conducted by the GfK-ISL Survey Institute. In total, 27,653 people aged 15 to 85 living in continental France were interviewed through CATI between October 2009 and July 2010. 

The design was a two-stage random sampling stratified by size of urban area and geographical area: the first stage of the sampling involved selecting households by using random digit dialling (RDD). The second stage involved selecting a member in the household through the Kish method [3], without replacement in case of refusal (neither at the household, nor at the individual level). In this way, 24,709 people contacted through their landline phones were interviewed. RDD can “catch” households that do not appear in the directory. These households represent about 30% of French households, and these individuals are more likely to smoke than people registered in the directory, the difference being partly explained by their different socioeconomic structures [4,5]. In addition, previous studies have shown a higher prevalence in individuals from households with a cell phone and without a landline than in the others [6,7]. In France, these “mobile-only” households account for 12% of all households. An additional sample of 2,944 mobile-only individuals were interviewed using the same process of generation and selection as for the landline phone sample.

Specific efforts were made to successfully reach households: up to 40 attempts were made for each generated phone number, since it has already been shown that “hard-to-reach” people, like non-respondents, have specific health behaviour [8-12]. As well, people who firstly refused to participate were contacted a second time by specially trained interviewers. This protocol allowed to recover 20% of the final sample. Details of the survey methodology have been previously published elsewhere [13-15].

### Quota survey

To assess comparability between random sampling and quota sampling, another telephone survey was conducted between September and December 2010 by the BVA Survey Institute on behalf of the French Institute for Health Promotion and Health Education. In total, 8,018 people aged 15 to 85 years-old were interviewed. The design was a quota sampling based on the French population structure in terms of gender crossed by age, and occupation of the head of the household, after stratification by size of urban area and geographical area. Reference data came from the 2008 Employment Survey, which is based on the French population census.

Since the quota method is designed with the only aim of having final margins equal to those of a defined population, French quota surveys are generally conducted on samples of landline telephone numbers randomly selected from the main French landline directory. However, mainly because young adults are more and more difficult to reach in the context of telephone surveys, several survey agencies, including BVA, have added a cell random digit-dialling sample (whether individuals also have a landline phone or not). In this study, “cell-only” households ended up representing 6% of the whole sample. Nevertheless, unlike the Health Barometer Survey, households with unlisted landline phone numbers were not included in the quota sampling.

Moreover, in the quota survey, phone numbers with no answer at the first call or a refusal by the person answering were not dialled again and were replaced. Lastly, in the quota sampling, there was no random selection within the household. The interviewee was either the person who hung up if he/she met the quota variables or any person meeting these selection criteria in the household.

### Variables

Sociodemographic variables were collected in both random and quota sample surveys: age, gender, geographical region, size of urban area (rural village, fewer than 20,000 inhabitants, 20,000 to 100,000 inhabitants, 100,000 to 200,000 inhabitants, more than 200,000 inhabitants, urban area of Paris), level of education (less than high school, high school graduate, college graduate) and occupational status (employed, student, unemployed, retired, other non-working).

Smoking status (current smoker or daily smoker) was assessed using two questions: “do you smoke tobacco, at least occasionally?”, and (if yes) “do you smoke every day?”

Given the survey period was different in each survey, the year and month when smokers had started to smoke and the year and month when former smokers had quit were asked in the quota survey. Thus, smoking status in January 2010 could be assessed at a time when half of people had been interviewed as part of the Health Barometer.

### Analysis

The analysis was carried out on 25,990 individuals from the Health Barometer and 7,621 from the quota survey all aged 15 to 75, the age group on which evolution of smoking prevalence has been assessed in France until now. Data from both surveys have been weighted according to gender crossed by age, size of urban area, geographical region and level of education. Distinct weights were calculated in the Health Barometer for the whole sample, landline phone owners, listed numbers. The 2008 French Employment Survey was used as a reference for margins.

First, both surveys were compared in terms of socioeconomic characteristics: gender, age, size of urban area, level of diploma and occupational status. Socioeconomic data were also crossed with the phone equipment and the needed number of calls in the Health Barometer. Crude and weighted percentages are shown. Next, prevalence of current and daily tobacco smoking was assessed in both surveys: in the whole sample (whether they have a landline and/or a cell phone) and among landline phone owners (with or without a cell phone) in the quota survey; in the whole sample, among landline phone owners and among people listed in the telephone directory in the Health Barometer. Design-based Pearson chi-square tests with Rao-Scott second order correction were used to test differences in proportions. Differences in prevalence according to the phone equipment and the survey mode were controlled using logistic regressions adjusted for sociodemographic variables. Lastly, in order to explain potential differences, the prevalence obtained according to the needed number of calls was estimated in the Health Barometer among landline phone owners, and was modelled using logistic regression adjusted for gender, age, level of education, occupational status and kind of phone equipment.

## Results

### Sociodemographic characteristics of the samples

Before weighting, males are under-represented in the Health Barometer compared with the quota survey (44.9% vs 48.5%, p-value<0,001) (Table 1). Undergraduate respondents are less represented in the quota survey (41.3%) than in the random survey (50.1%, p-value<0,001). Besides, after weighting, unemployed people are about twice as frequent in the Health Barometer (8.2%) as in the quota survey (4.5%, p-value<0,001).

**Table 1:** Socioeconomic characteristics of the 15-75 years-old interviewed in the quota survey and in the Health Barometer (HB), in the whole, among landline phone owners and among listed respondents in the Health Barometer.

	**Quota (N=7,621)**		**HB (N=25,990)**		**HB landline phone (N=21,952)**		**HB listed (N=11,433)**	
	crude %	weighted %	crude %	weighted %	crude %	weighted %	crude %	weighted %
**Gender**								
Male	48,5%	48,6%	44,9%	48,8%	43,9%	48,8%	44,6%	48,9%
Female	51,5%	51,5%	55,1%	51,2%	56,1%	51,2%	55,4%	51,1%
**Age**								
15-24 years old	12,4%	16,3%	13,2%	16,7%	11,0%	16,8%	9,8%	17,1%
25-34 years old	15,7%	16,6%	16,5%	16,7%	13,6%	16,6%	11,1%	16,4%
35-44 years old	22,6%	18,7%	20,7%	19,0%	20,8%	19,0%	18,6%	19,1%
45-54 years old	20,1%	18,3%	17,8%	18,6%	18,7%	18,6%	19,0%	18,6%
55-64 years old	16,8%	16,6%	19,4%	16,8%	21,6%	16,8%	23,9%	16,8%
65-75 years old	12,4%	13,4%	12,4%	12,2%	14,3%	12,1%	17,5%	12,0%
**Size of urban area**								
Rural	28,3%	26,0%	27,7%	25,8%	30,1%	25,9%	37,8%	26,2%
Less than 20,000 inhab.	16,3%	17,5%	18,8%	17,3%	19,3%	17,3%	20,7%	17,5%
20,000 to 100,000 inhab.	11,8%	12,7%	12,5%	12,7%	12,1%	12,6%	12,0%	12,7%
100,000 to 200,000 inhab.	5,7%	5,6%	5,1%	5,6%	4,8%	5,6%	4,3%	5,5%
More than 200,000 inhab.	22,4%	22,0%	21,9%	22,4%	20,4%	22,3%	16,5%	22,1%
Urban area of Paris	15,5%	16,2%	14,0%	16,3%	13,2%	16,2%	8,7%	15,9%
**Diploma**								
Less than high school	41,3%	60,0%	50,1%	59,8%	49,9%	59,6%	52,1%	59,5%
High school graduate	18,3%	17,7%	18,2%	17,6%	17,9%	17,7%	17,2%	17,7%
College graduate	40,4%	22,4%	31,7%	22,6%	32,2%	22,7%	30,7%	22,8%
**Occupational status**								
Employed	60,6%	55,2%	57,0%	54,2%	56,4%	55,5%	53,8%	55,9%
Student	8,9%	11,7%	8,9%	11,4%	8,2%	11,9%	7,1%	11,9%
Unemployed	3,9%	4,5%	7,1%	8,2%	5,7%	7,0%	5,0%	6,7%
Retired	20,6%	21,6%	21,2%	19,5%	24,2%	19,6%	28,5%	19,8%
Other non-working	6,0%	6,9%	5,8%	6,7%	5,5%	5,9%	5,5%	5,7%

Crude and weighted percentages.

With regard to the type of phone equipment in the 2010 Health Barometer, there are more individuals aged 55 years old or more among the ones who are listed in the directory (41.4% vs 24.3% among unlisted respondents or cell-only ones, p-value<0,001). Respondents who are listed in the directory less often live in the urban area of Paris (8.7% vs 18.1% of the ones who live elsewhere, p-value<0,001) and more often live in rural areas (37.8% vs 19.8%, p-value<0,001). After weighting, unemployed people are less represented among landline phone owners (5.7% vs 16.3% of the ones who only have a cell phone, p-value<0,001).

In the 2010 Health Barometer, “hard-to-reach” respondents are more frequently males: 53.3% of the respondents who were interviewed after more than 20 calls are males vs 44.2% of the ones for whom one or two calls were sufficient. “Hard-to-reach” respondents were also younger, more often employed and had a higher level of education (Table 2).

**Table 2:** Socioeconomic characteristics of the 15-75 years-old interviewed in the Health Barometer according to the number of calls before the interview.

	**1-2 calls**	**3-5 calls**	**6-10 calls**	**11-20 calls**	**more than 20 calls**
**Gender**					
Male	44,2%	47,8%	51,8%	52,4%	53,3%
Female	55,8%	52,2%	48,3%	47,7%	46,8%
**Age**					
15-24 years old	11,2%	17,3%	20,6%	20,5%	18,7%
25-34 years old	14,6%	15,1%	15,8%	19,5%	23,9%
35-44 years old	17,6%	18,8%	19,4%	19,4%	22,4%
45-54 years old	17,9%	19,4%	18,7%	18,5%	18,3%
55-64 years old	20,1%	17,2%	16,2%	13,7%	12,2%
65-75 years old	18,7%	12,2%	9,4%	8,3%	4,5%
**Diploma**					
Less than high school	62,5%	60,2%	59,0%	57,0%	55,4%
High school graduate	17,2%	17,4%	17,9%	17,8%	19,2%
College graduate	20,3%	22,5%	23,2%	25,3%	25,4%
**Occupational status**					
Employed	47,7%	54,6%	57,2%	61,9%	67,8%
Student	8,2%	12,5%	14,5%	14,5%	12,2%
Unemployed	7,0%	7,2%	7,1%	6,5%	7,5%
Retired	28,7%	19,8%	16,2%	13,6%	8,6%
Other non-working	8,4%	5,9%	5,0%	3,6%	3,9%

Weighted percentages

### Prevalence of smoking according to the survey and the phone equipment

On the whole, the current smoking prevalence among 15–75 years old is 33.9% in the Health Barometer and 30.2% in the quota survey (test of difference of proportions: p-value<0.001) (Table 2). The daily smoking prevalence is 27.5% in the Health Barometer and 25.3% in the quota survey (p-value<0.01). 

Considering only landline phone owners, smoking prevalences are also higher in the random survey than in the quota survey (31.8% vs 28.9% for current smoking, p-value<0.001; 25.5% vs 24.0% for daily smoking, p-value<0.05). When keeping only the listed numbers, current smoking prevalence remains higher in the Health Barometer than in the quota survey (31.2% vs 28.9%, p-value<0.01) but not daily smoking prevalence (Table 3). The difference in current smoking prevalence is still significant when respondents from the Health Barometer converted to interview after initial refusal are excluded from the analysis (31.0% vs 28.9%, p-value<0.05).

**Table 3:** Prevalence of current and daily smoking in both surveys according to telephone equipment in 15–75 years-old.

	Health Barometer (HB)			Quota Survey	
	Whole sample^1^	Landline phone owners^2^	Listed numbers^3^	Whole sample	Landline phone owners
Prevalence of current smoking prevalence	33.9%*** [33.2-34.5]	31.8%*** [31.0-32.5]	31.2%** [30.0-32.4]	30.2% [29.1-31.4]	28.9% [27.7-30.0]
Prevalence of daily smoking	27.5%** [26.8-28.1]	25.5%* [24.8-26.2]	24.9%ns [23.7-26.0]	25.3% [24.2-26.4]	24.0% [22.9-25.1]

Note: 1 Comparison between the whole sample of HB and the whole sample of the quota survey

2 Comparison between landline phone owners of HB and landline phone owners of the quota survey

3 Comparison between listed numbers of HB and landline phone owners of the quota survey

Pearson’s chi-square test: *** p<0.001; ** p<0.01; * p<0.05; ns: not significant

Weighted percentages and 95% confidence intervals.

### Factors related with current and daily tobacco smoking

As shown in other studies [16], a low level of diploma and unemployment are related with current and daily tobacco smoking in the pooled database (Table 4). Cell-only owners are also more likely to smoke after adjusment for sociodemographics (ORa=1.7 [1.6-1.8] for current and daily smoking compared with landline phone owners). After adjustement for sociodemographics and phone equipment, an association with the survey mode (random vs quotas) remains for current smoking (ORa=1.1 [1.1-1.2] for the random survey compared with the quotas survey, p-value<0.001), but not for daily smoking. The odds-ratios do not change when the respondents who accepted to answer after initial refusal are removed from the logistic model

**Table 4:** Weighted percentages, adjusted odds-ratios (aOR) and 95% confidence intervals (CI) from logistic regressions on current and daily smoking among the whole of respondents (N=33,527).

	**Current smoking**			**Daily smoking**		
	%	aOR	95% CI	%	aOR	95% CI
**Gender**	***			***		
Male (ref.) (n=15372)	36.8	- 1 -		30.0	- 1 -	
Female (n=18239)	29.5	0.7***	[0.7-0.8]	24.1	0.8***	[0.7-0.8]
**Age**	***			***		
15-24 years-old (ref.) (n=4377)	38.7	- 1 -		27.9	- 1 -	
25-34 years-old (n=5492)	47.0	1.0	[0.9-1.2]	39.1	1.1*	[1.0-1.3]
35-44 years-old (n=7087)	40.5	0.8***	[0.7-0.9]	34.3	0.9	[0.8-1.0]
45-54 years-old (n=6172)	34.5	0.6***	[0.6-0.7]	29.6	0.8***	[0.7-0.8]
55-64 years-old (n=6322)	20.7	0.4***	[0.3-0.4]	17.3	0.4***	[0.4-0.5]
65-75 years-old (n=4161)	9.7	0.2***	[0.2-0.2]	7.4	0.2***	[0.2-0.3]
**Diploma**	***			***		
Less than high school (ref.) (n=16128)	34.0	- 1 -		28.9	- 1 -	
High school graduate (n=6123)	34.2	0.9***	[0.8-0.9]	27.2	0.8***	[0.8-0.9]
College graduate (n=11294)	29.7	0.7***	[0.7-0.7]	21.8	0.6***	[0.6-0.6]
**Occupational status**	***			***		
Employed (ref.) (n=19427)	37.7	- 1 -		31.2	- 1 -	
Student (n=2995)	31.2	0.6***	[0.5-0.6]	20.0	0.5***	[0.4-0.5]
Unemployed (n=2131)	53.8	1.5***	[1.4-1.7]	47.3	1.6***	[1.4-1.7]
Retired (n=7067)	12.9	0.7***	[0.7-0.8]	10.5	0.8***	[0.7-0.9]
Other non-working (n=1971)	35.6	1.1**	[1.0-1.3]	31.5	1.2***	[1.1-1.4]
**Cell-only**	***			***		
No (ref.) (n=30189)	30.2	- 1 -		24.4	- 1 -	
Yes (n=3422)	53.1	1.7***	[1.6-1.8]	45.3	1.7***	[1.6-1.8]
**Survey mode**	***			**		
Quotas (ref.) (n=7621)	30.2	- 1 -		25.3	- 1 -	
Random (n=25990)	33.9	1.1***	[1.1-1.2]	27.5	1.0	[1.0-1.1]

Pearson’s chi-square test for percentages, Wald test for odds-ratios : *** p<0.001 ; ** p<0.01 ; * p<0.05

As expected, in the model restricted to landline phone owners interviewed in the Health Barometer, being unlisted in the directory is related to a higher risk of being a smoker or a daily smoker (ORa=1.2 [1.1-1.3], p-value<0.001) after adjustment for gender, age, level of diploma and occupational status (Table 5).

**Table 5:** Weighted percentages, adjusted odds-ratios (aOR) and 95% confidence intervals (CI) from logistic regressions on current and daily smoking among the Health Barometer landline phone owners (N=21,884).

	**Current smoking**			**Daily smoking**		
	%	aOR	95% CI	%	aOR	95% CI
**Gender**	***			***		
Male (ref.) (n=9630)	35,6	- 1 -		28,6	- 1 -	
Female (n=12322)	28.1	0.7***	[0.7-0.8]	22.5	0.8***	[0.7-0.8]
**Age**	***			***		
15-24 years-old (ref.) (n=2414)	37.8	- 1 -		26.2	- 1 -	
25-34 years-old (n=2980)	45.3	1.0	[0.8-1.1]	37.4	1.1	[0.9-1.3]
35-44 years-old (n=4558)	39.6	0.7***	[0.6-0.8]	32.9	0.8*	[0.7-1.0]
45-54 years-old (n=4110)	32.3	0.5***	[0.5-0.6]	27.5	0.7***	[0.6-0.8]
55-64 years-old (n=4752)	19.2	0.3***	[0.3-0.4]	16.0	0.4***	[0.3-0.5]
65-75 years-old (n=3138)	9.2	0.2***	[0.1-0.2]	6.7	0.2***	[0.2-0.3]
**Diploma**	***			***		
Less than high school (ref.) (n=10927)	32.2	- 1 -		27.2	- 1 -	
High school graduate (n=3925)	34.3	0.9	[0.9-1.0]	26.4	0.8***	[0.8-0.9]
College graduate (n=7044)	28.8	0.7***	[0.7-0.8]	20.2	0.6***	[0.5-0.6]
**Occupational status**	***			***		
Employed (ref.) (n=12378)	36.8	- 1 -		30.2	- 1 -	
Student (n=1796)	31.0	0.5***	[0.4-0.6]	19.5	0.4***	[0.3-0.5]
Unemployed (n=1253)	51.0	1.5***	[1.3-1.7]	44.3	1.6***	[1.4-1.8]
Retired (n=5308)	12.1	0.7***	[0.6-0.8]	9.4	0.7***	[0.6-0.8]
Other non-working (n=1204)	28.2	1.0	[0.9-1.2]	24.7	1.1	[0.9-1.2]
**Type of phone line**	***			***		
Listed number (réf.) (n=11433)	29.2	- 1 -		23.2	- 1 -	
Listed number except for direct marketing (n=2745)	31.2	0.9	[0.8-1.0]	23.4	0.9**	[0.8-1.0]
Unlisted number (n=7774)	35.4	1.2***	[1.1-1.3]	29.2	1.2***	[1.1-1.3]
**Number of calls needed**	***			***		
1-2 (ref.) (n=6733)	28.2	- 1 -		22.5	- 1 -	
3-5 (n=5844)	30.7	1.0	[0.9-1.1]	24.6	1.0	[0.9-1.1]
6-10 (n=4251)	32.9	1.0	[0.9-1.1]	26.4	1.0	[0.9-1.1]
11-20 (n=2861)	35.1	1.1	[1.0-1.2]	27.6	1.1	[0.9-1.2]
>20 (n=2263)	37.4	1.1*	[1.0-1.3]	31.2	1.2*	[1.0-1.3]
**Interview after initial refusal**						
No (ref.) (n=17462)	31.8	- 1 -		25.3	- 1 -	
Yes (n=4490)	31.5	1	[0.9-1.1]	25.9	1.0	[0.9-1.1]

Pearson’s chi-square test for percentages, Wald test for odds-ratios : *** p<0.001 ; ** p<0.01 ; * p<0.05

The prevalence of current and daily tobacco smoking also dramatically increases with the number of calls needed before the interview is completed. Prevalence is about ten points higher for both indicators between people interviewed after just one or two calls and those for whom more than twenty calls were needed. Even though this association is strongly mediated by socioeconomic characteristics, a small effect of the needed number of calls remains after adjustment for the hardest-to-reach people, in particular above twenty calls (ORa=1.1 [1.0-1.3] for current smoking compared with people interviewed after just one or two calls, p<0.05; ORa=1.2 [1.0-1.3] for daily smoking, p-value<0.05). On the other hand, no relation was shown for people who accepted to answer after initial refusal at the household level. 

## Discussion / Conclusion

Facing the tobacco epidemic, monitoring trends in tobacco use is a crucial issue worldwide [17,18]. This issue raised major methodological concerns. On the one hand, former studies have shown how far estimates from different methodologies could differ within several countries [19]. On the other hand, a previous analysis carried out in 1995 showed no significant difference between random and quota sampling for tobacco smoking prevalence in France [20]. In our study, tobacco smoking prevalence as assessed in the Health Barometer is markedly higher than the one obtained in the quota sampling survey. The difference is almost four points for current smoking (33.9% vs. 30.2%) and more than two points for daily smoking (27.5% vs. 25.3%). When restricting the analysis to the listed landline phone owners, a one-point difference remains for current smoking, but not for daily smoking.

Even if a 2-point difference in daily smoking appears to be slight, it corresponds to the significant increase in smoking prevalence that was observed between 2005 and 2010 in France with the Health Barometer [1], and it must be taken into consideration. The insistence coefficient, i.e., the number of calls needed to contact hard-to-reach populations, could partly explain the observed differences.

Most authors agree that increased reporting of substance use is a sign of improved validity in the methodology, because these behaviors tend to be underreported [21-27]. Thus, random sampling appears to be now more effective than quota sampling, by making it possible to interview hard-to-reach populations, whose proportion is currently increasing in France [28] and who are more often smokers than the ones who easily answer to surveys. On the whole, some specific populations are under-represented in standard quotas surveys, for example: people with a low level of education, unemployed people... Furthermore, due to the recent evolution in telephony, this analysis confirms the need to include from now on, in prevalence survey sampling, individuals who are impossible to contact in classic phone quota surveys, i.e. mobile-only owners, unlisted landline owners, etc. Lastly, random surveys covering the whole population are needed to enable accurate international comparisons [19].

A monitoring system for smoking prevalence based on quota surveys would assume that their biases are constant from one period to another, which would mean that hard-to-reach populations have the same trend in prevalence as the rest of the population. Another possibility is to set up a quota survey including an insistence coefficient in order to include a number of hard-to-reach people. 

To conclude, the present analysis by itself does not completely allow to decide on the possibility to use quotas surveys in order to monitor tobacco smoking prevalence. A new comparison between random and quotas survey is needed to see if the evolutions observed in both systems are consistent.
